# Supplementary material for: Isonicotinylation is a histone mark induced by the anti-tuberculosis first-line drug isoniazid
Source: Nat Commun. 2021 Sep 20;12:5548. doi: 10.1038/s41467-021-25867-y (PMC8452692; doi:10.1038/s41467-021-25867-y)
Supplement: Supplementary file 1 — Supplementary Information [file 41467_2021_25867_MOESM1_ESM.pdf]

# Isonicotinylation is a histone mark induced by the anti-tuberculosis first-line drug isoniazid

## Supplementary information

**Supplementary Table 1 The sequences for real-time RT-PCR primer pairs**

**Supplementary Fig. 1 The discovery of a +105.0215 Da mass shift**

**Supplementary Fig. 2 Identification and characterization of histone isonicotinylation**

**Supplementary Fig. 3 INH stimulates histone $K_{inic}$ in cells and mice**

**Supplementary Fig. 4 CBP/P300 function as isonicotinyltransferases and HADC3 as deisonicotinylase for histone $K_{inic}$**

**Supplementary Fig. 5 INH-induced histone isonicotinylation varies in tumours and upregulated cancer-related signalling pathway.**

**Supplementary Table 1 The sequences for real-time RT-PCR primer pairs**

| | |
|---|---|
| *GAPDH*: | 5'-CAGCAAGAGCACAAGAGGAA-3'（sense） |
| | 5'-CCCCTCTTCAAGGGGTCTAC-3'（antisense） |
| *Sat-2*: | 5'-CATCGAATGGAAATGAAAGGAGTC-3'（sense） |
| | 5'-ACCATTGGATGATTGCAGTCAA-3'（antisense） |
| *PIK3R1*: | 5'-TGGACGGCGAAGTAAAGCATT-3'（sense） |
| | 5'-AGTGTGACA TTGAGGGAGTCG-3'（antisense） |
| *CAMK4*: | 5'-GCCTCGTCCCGGATTACTG-3'（sense） |
| | 5'-TCCCCTTCTGTTTGCATCTGT-3'（antisense） |
| *CACNA1D*: | 5'-GCGAACGAGGCAAACTATG-3'（sense） |
| | 5'-TTGGAGCTATTCGGCTGAGAA-3'（antisense） |
| *CR2*: | 5'-GGTCCTCGGGATTTCTTGTGG-3'（sense） |
| | 5'-GAACAACTGTACCTTATCACGGT-3'（antisense） |
| *PLAU*: | 5'-GGGAATGGTCACTTTTACCGAG-3'（sense） |
| | 5'-GGGCATGGTACGTTTGCTG-3'（antisense） |
| *IL1B*: | 5'-ATGATGGCTTATTACAGTGGCAA-3'（sense） |
| | 5'-GTCGGAGATTCGTAGCTGGA-3'（antisense） |

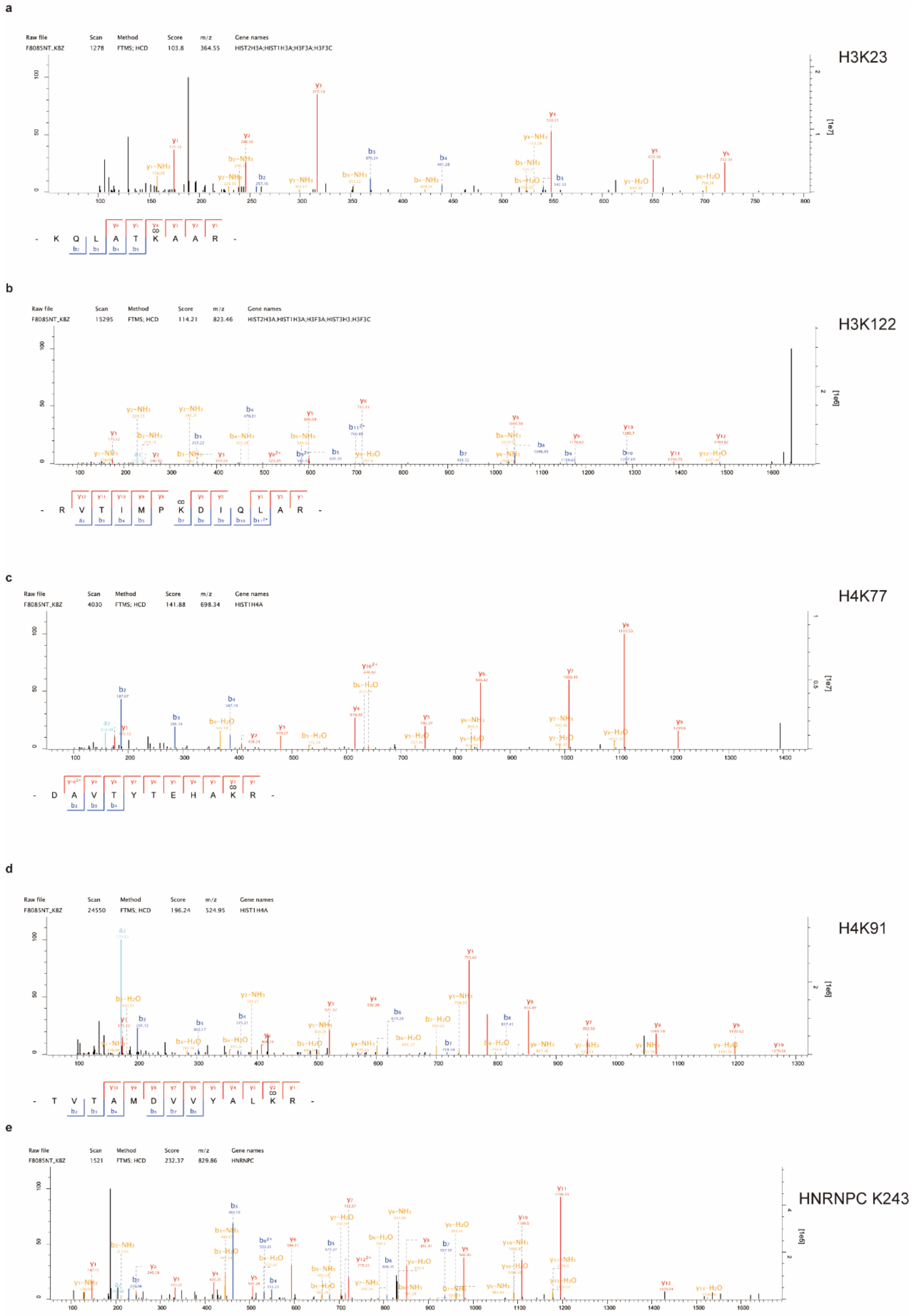

a
Raw file Scan Method Score m/z Gene names
F8085NT_KBZ 1278 FTMS; HCD 103.8 364.55 HIST2H3A;HIST1H3A;H3F3A;H3F3C
H3K23
- K Q L A T K A A R -
b
Raw file Scan Method Score m/z Gene names
F8085NT_KBZ 15295 FTMS; HCD 114.21 823.46 HIST2H3A;HIST1H3A;H3F3A;HIST3H3;H3F3C
H3K122
- R V T I M P K D I Q L A R -
c
Raw file Scan Method Score m/z Gene names
F8085NT_KBZ 4030 FTMS; HCD 141.88 698.34 HIST1H4A
H4K77
- D A V T Y T E H A K R -
d
Raw file Scan Method Score m/z Gene names
F8085NT_KBZ 24550 FTMS; HCD 196.24 524.95 HIST1H4A
H4K91
- T V T A M D V V Y A L K R -
e
Raw file Scan Method Score m/z Gene names
F8085NT_KBZ 1521 FTMS; HCD 232.37 829.86 HNRNPC
HNRNPC K243

f

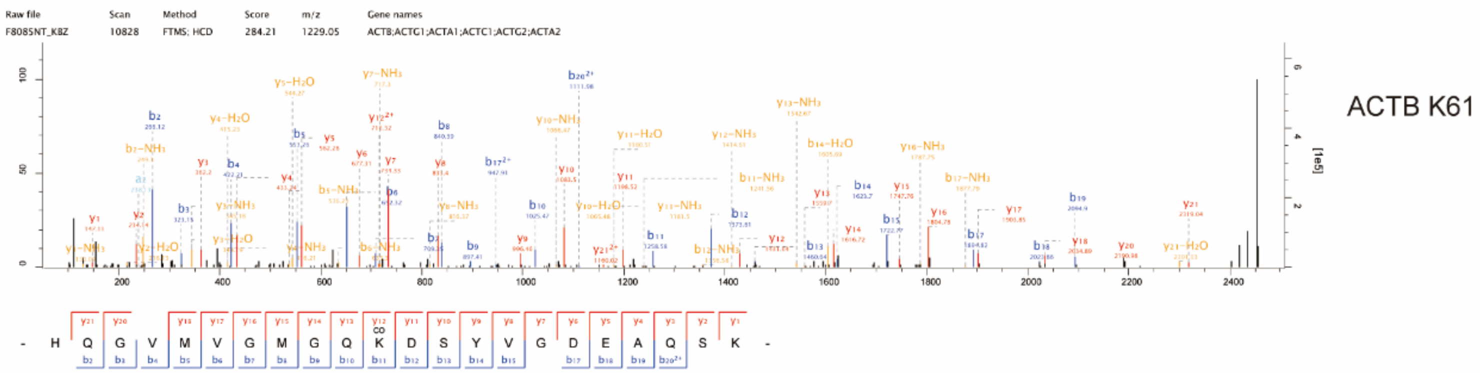


g

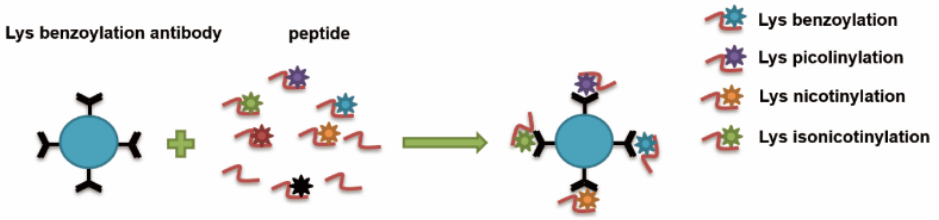


**Supplementary Fig. 1 The discovery of a +105.0215 Da mass shift**

**(a)** The spectrum of the charged ion (m/z 364.55) indicated that a mass shift of +105.0215Da at the H3K23 position in the peptide KQLATK$_{+105.0215}$AAR.

**(b)** The spectrum of the charged ion (m/z 823.46) indicated that a mass shift of +105.0215Da at the H3K122 position in the peptide RVTIMPK$_{+105.0215}$DIQLAR.

**(c)** The spectrum of the charged ion (m/z 698.34) indicated that a mass shift of +105.0215Da at the H4K77 position in the peptide DAVTYTEHAK$_{+105.0215}$R.

**(d)** The spectrum of the charged ion (m/z 524.95) indicated that a mass shift of +105.0215Da at the H4K91 position in the peptide TVTAMDVVYALK$_{+105.0215}$R.

**(e)** The spectrum of the charged ion (m/z 829.86) indicated that a mass shift of +105.0215Da at the HNRNPC K243 position in the peptide NDK$_{+105.0215}$SEEEQSSSSVK.

**(f)** The spectrum of the charged ion (m/z 1229.05) indicated that a mass shift of +105.0215Da at the ACTB K61 position in the peptide HQGVMVGMGQK$_{+105.0215}$DSYVGDEAQSK. The b ions indicate the fragmentation ions containing the N-terminus of the peptide and the y ions are the fragmentation ions containing the C-terminus of the peptide.

**(g)** Chematic diagram of the antibody against lysine benzoylation can be used to enrich for peptides with structurally-related acylations (lysine picolinylation, lysine nicotinylation, lysine isonicotinylation).

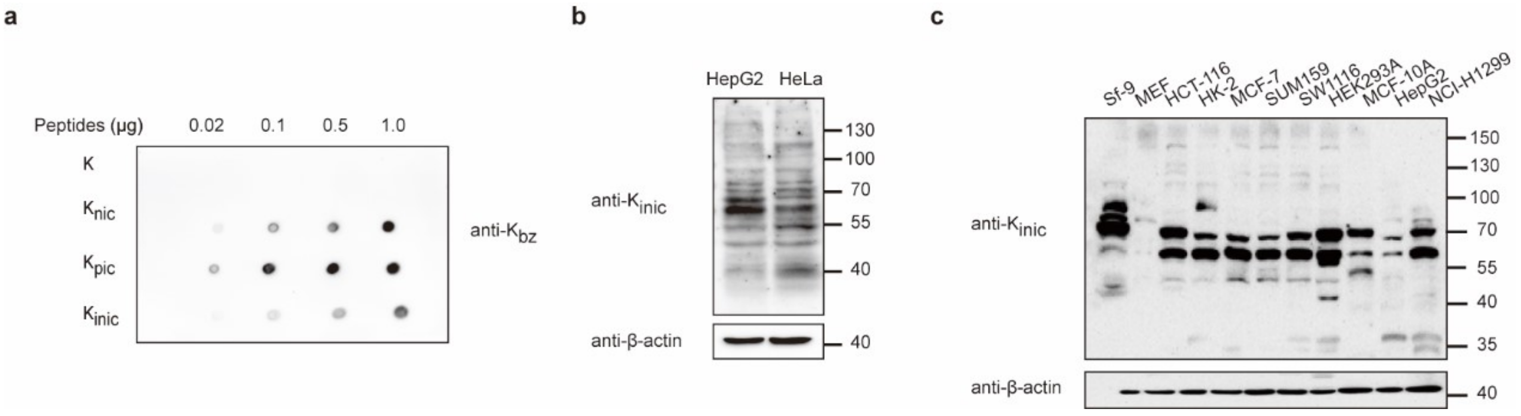


**Supplementary Fig. 2 Identification and characterization of histone isonicotinylation**

**(a)** Dot-blot assay was used to verify the specificity of pan antibody against $K_{bz}$ modification. Nitrocellulose membrane was spotted by different amounts of unmodified peptide (DAVTYTEHAKR), lysine nicotinylated peptide (DAVTYTEHA$K_{nic}$R), lysine picolinylated peptide (DAVTYTEHA$K_{pic}$R), lysine isonicotinylated peptide (DAVTYTEHA$K_{inic}$R) and detected with the pan-$K_{bz}$ antibody.

**(b)** Detection of non-histone $K_{inic}$ mark in HepG2 and HeLa cells. Total proteins extracted from HepG2 and HeLa cells were tested using pan-$K_{inic}$ and β-actin antibodies by Western blot.

**(c)** Detection of non-histone $K_{inic}$ mark in Sf-9, MEF, HCT-116, HK-2, MCF-7, SUM159, SW1116, HEK293A, MCF-10A, HepG2, NCI-H1299 cells. Total proteins extracted from these cells were tested using pan-$K_{inic}$ and β-actin antibodies by Western blot analysis.

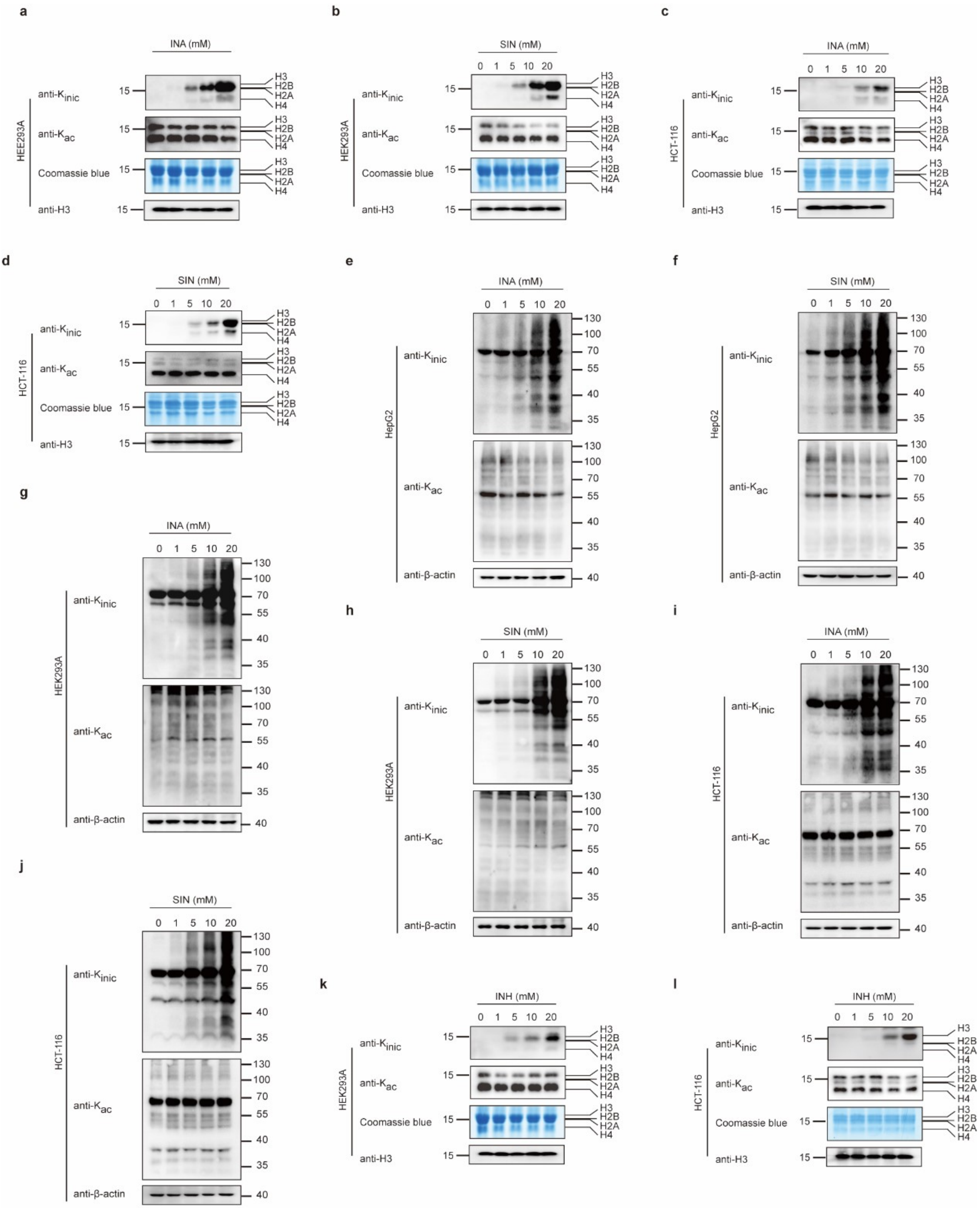
a
INA (mM)
anti-Kinic
anti-Kac
Coomassie blue
anti-H3
HEE293A
H3
H2B
H2A
H4
15
b
SIN (mM)
0 1 5 10 20
HEK293A
c
INA (mM)
0 1 5 10 20
HCT-116
d
SIN (mM)
0 1 5 10 20
HCT-116
e
INA (mM)
0 1 5 10 20
HepG2
anti-β-actin
130
100
70
55
40
35
f
SIN (mM)
0 1 5 10 20
HepG2
g
INA (mM)
0 1 5 10 20
HEK293A
h
SIN (mM)
0 1 5 10 20
HEK293A
i
INA (mM)
0 1 5 10 20
HCT-116
j
SIN (mM)
0 1 5 10 20
HCT-116
k
INH (mM)
0 1 5 10 20
HEK293A
l
INH (mM)
0 1 5 10 20
HCT-116

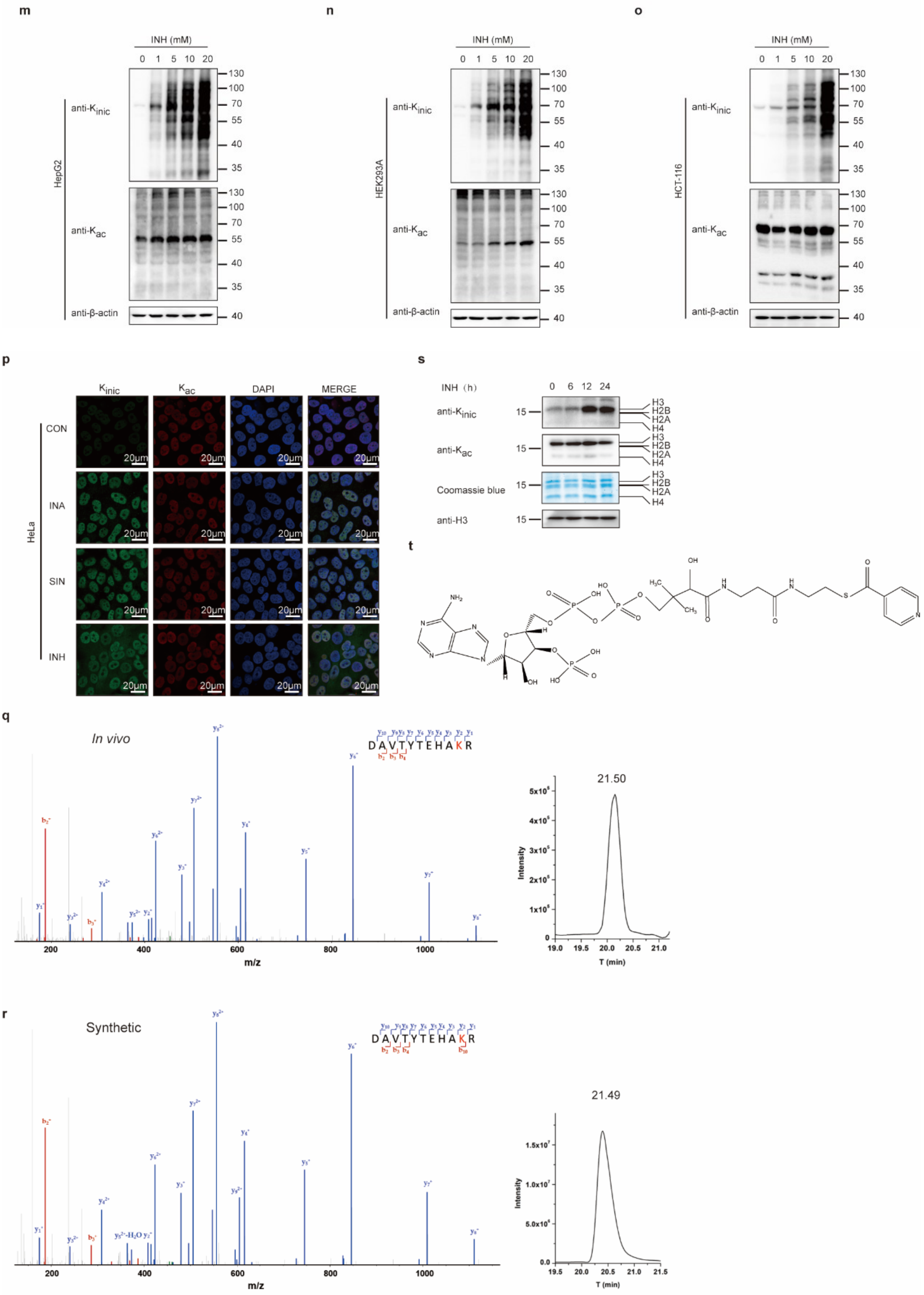
m
n
o
INH (mM)
0 1 5 10 20
130
100
70
55
40
35
anti-K$_{inic}$
anti-K$_{ac}$
anti-β-actin
HepG2
HEK293A
HCT-116
p
K$_{inic}$
K$_{ac}$
DAPI
MERGE
CON
INA
SIN
INH
HeLa
20μm
s
INH（h）
0 6 12 24
anti-K$_{inic}$
anti-K$_{ac}$
Coomassie blue
anti-H3
15
H3
H2B
H2A
H4
t
q
In vivo
DAVTYTEHAKR
m/z
Intensity
T (min)
21.50
r
Synthetic
21.49

**Supplementary Fig. 3 INH stimulates histone $K_{inic}$ in cells and mice**

**(a-p)** INA, SIN and INH stimulated histone and non-histone $K_{inic}$.

**(a)** Core histones acid-extracted from HEK293A cells that treated with increasing concentration of INA for 24 h and tested using pan-$K_{inic}$, pan-$K_{ac}$ and H3 antibodies by Western blot analysis. Total histones were visualized with Coomassie blue staining.

**(b)** Core histones acid-extracted from HEK293A cells that treated with increasing concentration of SIN for 24 h and tested using pan-$K_{inic}$, pan-$K_{ac}$ and H3 antibodies by Western blot analysis. Total histones were visualized with Coomassie blue staining.

**(c)** Core histones acid-extracted from HCT-116 cells that treated with increasing concentration of INA for 24 h and tested using pan-$K_{inic}$, pan-$K_{ac}$ and H3 antibodies by Western blot analysis. Total histones were visualized with Coomassie blue staining.

**(d)** Core histones acid-extracted from HCT-116 cells that treated with increasing concentration of SIN for 24 h and tested using pan-$K_{inic}$, pan-$K_{ac}$ and H3 antibodies by Western blot analysis. Total histones were visualized with Coomassie blue staining.

**(e)** Total proteins extracted from HepG2 cells that treated with increasing concentration of INA for 24 h and tested using pan-$K_{inic}$, pan-$K_{ac}$ and β-actin antibodies by Western blot analysis.

**(f)** Total proteins extracted from HepG2 cells that treated with increasing concentration of SIN for 24 h and tested using pan-$K_{inic}$, pan-$K_{ac}$ and β-actin antibodies by Western blot analysis.

**(g)** Total proteins extracted from HEK293A cells that treated with increasing concentration of INA for 24 h and tested using pan-$K_{inic}$, pan-$K_{ac}$ and β-actin antibodies by Western blot analysis.

**(h)** Total proteins extracted from HEK293A cells that treated with increasing concentration of SIN for 24 h and tested using pan-$K_{inic}$, pan-$K_{ac}$ and β-actin antibodies by Western blot analysis.

**(i)** Total proteins extracted from HCT-116 cells that treated with increasing concentration of INA for 24 h and tested using pan-$K_{inic}$, pan-$K_{ac}$ and β-actin antibodies by Western blot analysis.

**(j)** Total proteins extracted from HCT-116 cells that treated with increasing concentration of SIN for 24 h and tested using pan-$K_{inic}$, pan-$K_{ac}$ and β-actin antibodies by Western blot analysis.

**(k)** Core histones acid-extracted from HEK293A cells that treated with increasing concentration of INH for 24 h and tested using pan-$K_{inic}$, pan-$K_{ac}$ and H3 antibodies by Western blot analysis. Total histones were visualized with Coomassie blue staining.

**(l)** Core histones acid-extracted from HCT-116 cells that treated with increasing concentration of INH for 24 h and tested using pan-$K_{inic}$, pan-$K_{ac}$ and H3 antibodies by Western blot analysis. Total histones were visualized with Coomassie blue staining.

**(m)** Total proteins extracted from HepG2 cells that treated with increasing concentration of INH for 24 h and tested using pan-$K_{inic}$, pan-$K_{ac}$ and β-actin antibodies by Western blot analysis.

**(n)** Total proteins extracted from HEK293A cells that treated with increasing concentration of INH for 24 h and tested using pan-$K_{inic}$, pan-$K_{ac}$ and β-actin antibodies by Western blot analysis.

**(o)** Total proteins extracted from HCT-116 cells that treated with increasing concentration of INH for 24 h and tested using pan-$K_{inic}$, pan-$K_{ac}$ and β-actin antibodies by Western blot analysis.

**(p)** HeLa cells were treated with 10 mM INA, SIN and INH respectively for 24 h and stained with pan-$K_{inic}$ rabbit (green) and pan-$K_{ac}$ mouse (red) antibodies. Nuclei were stained with DAPI (blue), followed by visualization with confocal microscopy. Scale bar, 20 μm.

**(q)** The MS/MS spectra (left) and the extracted ion chromatograms (right) of *in vivo* peptide of DAVTYTEHA$K_{inic}$R by HPLC-MS/MS analysis.

**(r)** The MS/MS spectra (left) and the extracted ion chromatograms (right) of synthetic peptide of DAVTYTEHA$K_{inic}$R by HPLC-MS/MS analysis.

**(s)** Core histones acid-extracted from HepG2 cells that treated with 10 mM INH for 0, 6, 12 or 24 h and tested using pan-$K_{inic}$, pan-$K_{ac}$ and H3 antibodies by Western blot analysis. Total histones were visualized with Coomassie blue staining.

**(t)** The chemical formula of isonicotinyl-CoA.

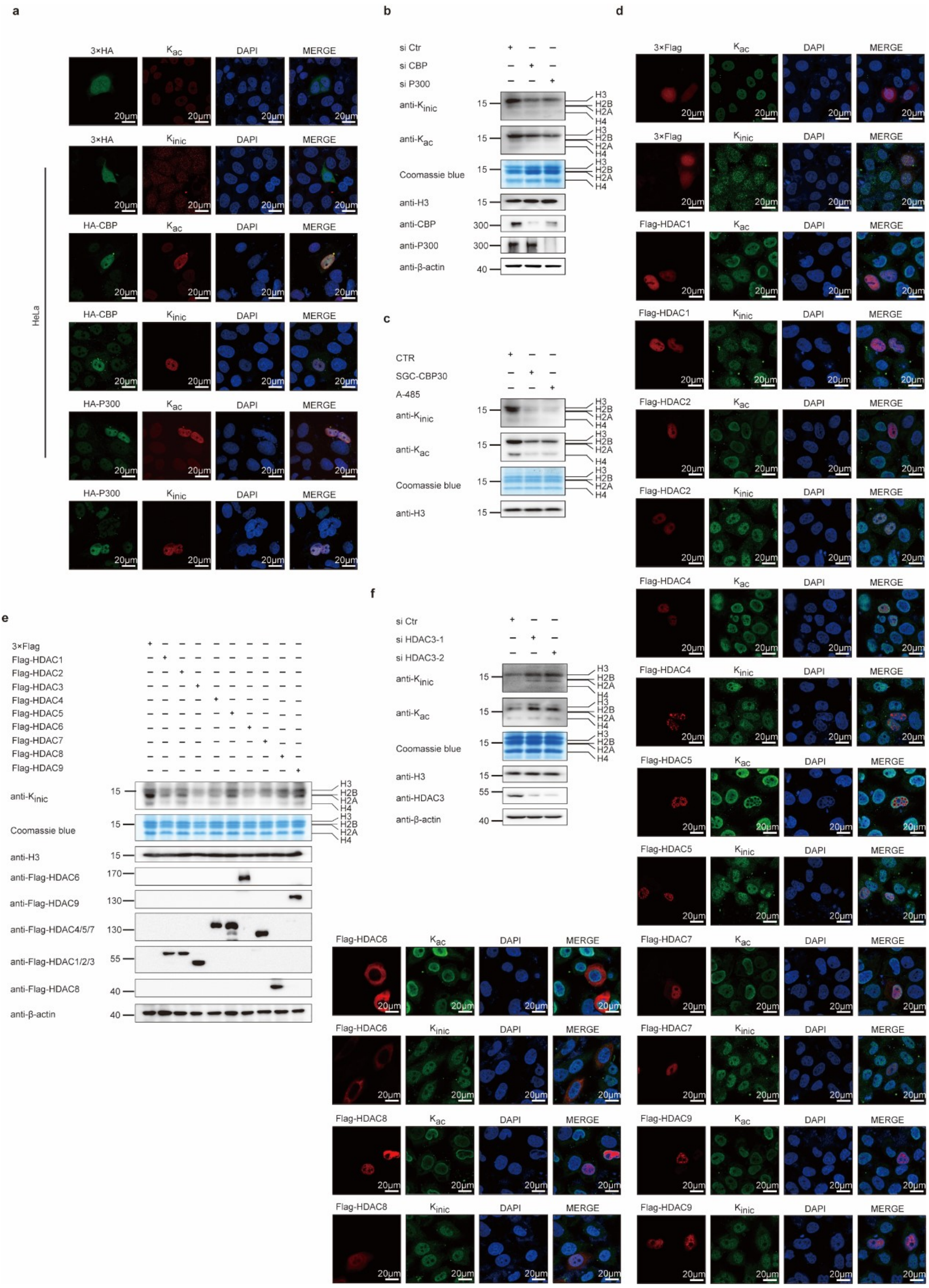
a
3×HA
Kac
Kinic
DAPI
MERGE
HA-CBP
HA-P300
HeLa
20μm
b
si Ctr
si CBP
si P300
anti-Kinic
anti-Kac
Coomassie blue
anti-H3
anti-CBP
anti-P300
anti-β-actin
15
300
40
H3
H2B
H2A
H4
c
CTR
SGC-CBP30
A-485
d
3×Flag
Flag-HDAC1
Flag-HDAC2
Flag-HDAC4
Flag-HDAC5
Flag-HDAC6
Flag-HDAC7
Flag-HDAC8
Flag-HDAC9
e
Flag-HDAC3
anti-Flag-HDAC6
anti-Flag-HDAC9
anti-Flag-HDAC4/5/7
anti-Flag-HDAC1/2/3
anti-Flag-HDAC8
170
130
55
f
si HDAC3-1
si HDAC3-2
anti-HDAC3

**Supplementary Fig. 4 CBP/P300 function as isonicotinyltransferases and HADC3 as deisonicotinylase for histone $K_{inic}$**

**(a)** HeLa cells were transfected with 3×HA，HA-CBP or HA-P300 plasmids and stained with pan-$K_{ac}$ or pan- $K_{inic}$ rabbit (red) and HA-tag mouse (green) antibodies. Nuclei were stained with DAPI (blue), followed by visualization with confocal microscopy. Scale bar, 20 μm.

**(b)** HepG2 cells were transfected with small interfering RNA (siRNA) against CBP and P300, core histones acid-extracted and tested using pan-$K_{inic}$, pan-$K_{ac}$ and H3 antibodies. Total histones were visualized with Coomassie blue staining. Total proteins were extracted and tested using CBP, P300 and β-actin antibodies by Western blot analysis.

**(c)** HepG2 cells were treated with 1 μM SGC-CBP30 or 3 μM A-485, core histones acid-extracted and tested using pan-$K_{inic}$, pan-$K_{ac}$ and H3 antibodies. Total histones were visualized with Coomassie blue staining. And total proteins were extracted and tested using CBP, P300 and β-actin antibodies by Western blot analysis.

**(d)** HepG2 cells were transfected with 3×Flag, Flag-HDAC1, 2, 4-9 plasmids and stained with pan-$K_{ac}$ antibody or pan-$K_{inic}$ rabbit (green) and Flag-tag mouse (red) antibodies. Nuclei were stained with DAPI (blue), followed by visualization with confocal microscopy. Scale bar, 20 μm.

**(e)** HepG2 cells were transfected with 3×Flag, Flag-HDAC1-9 plasmids, core histones acid-extracted and tested using pan-$K_{inic}$ and H3 antibodies. Total histones were visualized with Coomassie blue staining. Total proteins were extracted and tested using Flag-tag and β-actin antibodies by Western blot analysis.

**(f)** HepG2 cells were transfected with small interfering RNA (siRNA) against HDAC3, core histones acid-extracted and tested using pan-$K_{inic}$, pan-$K_{ac}$ and H3 antibodies. Total histones were visualized with Coomassie blue staining. Total proteins were extracted and tested using HDAC3 and β-actin antibodies by Western blot analysis.

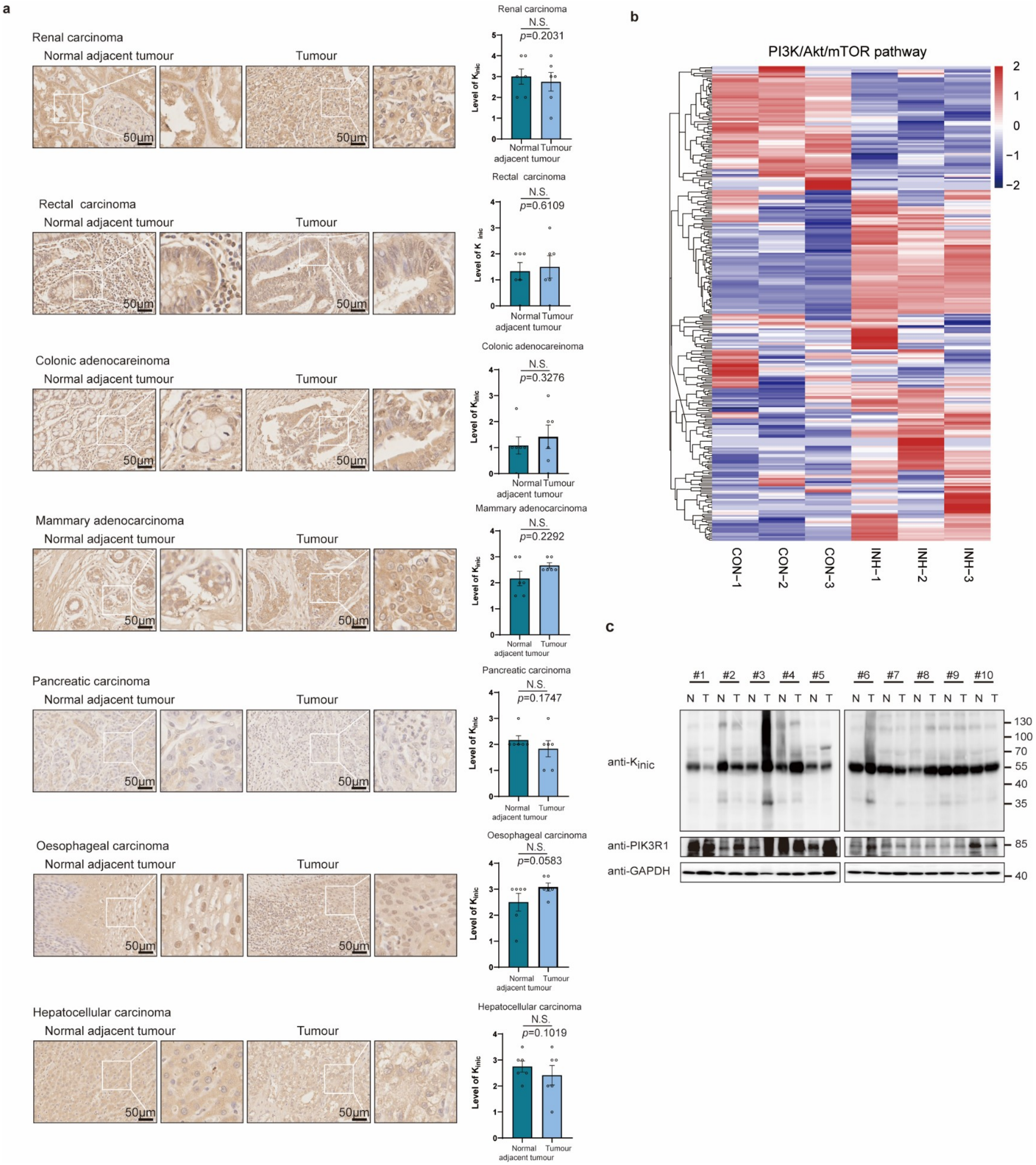


**Supplementary Fig. 5 INH-induced histone isonicotinylation varies in tumours and upregulated cancer-related signalling pathway.**

**(a)** Normal adjacent tumour tissues and tumour tissues of renal carcinoma, rectal carcinoma, colonic adenocareinoma, mammary adenocareinoma, pancreatic carcinoma, oesophageal carcinoma and hepatocellular carcinoma were performed by immunohistochemical staining with pan-$K_{inic}$ antibody respectively, followed by visualization with light microscopy. Scale bar, 50 μm. Quantifications of the scores of immunohistochemical staining (right) (n=6 samples, values were expressed as mean ±

SEM, as determined by paired two-tailed Student's *t*-test). **(b)** Heatmap of genes involved in PI3K/Akt/mTOR pathway in response to INH in RNA-seq data. "CON" indicates untreated HepG2 cells; "INH" indicates isoniazid treated HepG2 cells. Library sizes were normalized across samples, and the fragments per kilobasepermillion bases (FPKM) were centered and scaled in the row direction. **(c)** We randomly selected normal adjacent tumour tissues and tumour tissues of ten hepatic carcinoma patients, total proteins were extracted and tested using pan-$K_{inic}$, PIK3R1, and GAPDH antibodies by Western blot. N: normal adjacent tumour tissue; T: tumour tissue.
