# Supplementary material for: Isonicotinylation is a histone mark induced by the anti-tuberculosis first-line drug isoniazid
Source: Nat Commun. 2021 Sep 20;12:5548. doi: 10.1038/s41467-021-25867-y (PMC8452692; doi:10.1038/s41467-021-25867-y)
Supplement: Supplementary file 3 — Reporting Summary [file 41467_2021_25867_MOESM3_ESM.pdf]

nature research

Corresponding author(s): Hongquan Zhang

Last updated by author(s): Aug 31, 2021

Reporting Summary

Nature Research wishes to improve the reproducibility of the work that we publish. This form provides structure for consistency and transparency in reporting. For further information on Nature Research policies, see our Editorial Policies and the Editorial Policy Checklist.

Statistics

For all statistical analyses, confirm that the following items are present in the figure legend, table legend, main text, or Methods section.

n/a	Confirmed	
☐	☒	The exact sample size (*n*) for each experimental group/condition, given as a discrete number and unit of measurement
☐	☒	A statement on whether measurements were taken from distinct samples or whether the same sample was measured repeatedly
☐	☒	The statistical test(s) used AND whether they are one- or two-sided<br>*Only common tests should be described solely by name; describe more complex techniques in the Methods section.*
☒	☐	A description of all covariates tested
☒	☐	A description of any assumptions or corrections, such as tests of normality and adjustment for multiple comparisons
☐	☒	A full description of the statistical parameters including central tendency (e.g. means) or other basic estimates (e.g. regression coefficient) AND variation (e.g. standard deviation) or associated estimates of uncertainty (e.g. confidence intervals)
☐	☒	For null hypothesis testing, the test statistic (e.g. *F*, *t*, *r*) with confidence intervals, effect sizes, degrees of freedom and *P* value noted<br>*Give P values as exact values whenever suitable.*
☒	☐	For Bayesian analysis, information on the choice of priors and Markov chain Monte Carlo settings
☒	☐	For hierarchical and complex designs, identification of the appropriate level for tests and full reporting of outcomes
☒	☐	Estimates of effect sizes (e.g. Cohen's *d*, Pearson's *r*), indicating how they were calculated

Our web collection on statistics for biologists contains articles on many of the points above.

Software and code

Policy information about availability of computer code

Data collection	A Model 680 microplate reader was used for absorbance detected in a 96 well plates for BCA protein quantification. Tanon GIS Software (v1.100) was used for gel scanning. Real-time qPCR analysis: LightCycler 96 SW 1.1. SageCapture Imaging Software (v2.19.12.20190311) was used for SDS-PAGE scanning. A LSM-780 confocal laser-scanning microscope (with Zeiss confocal Zen Software (v2011) using a100´/1.4 NA objective lens) was used to capture images from immuno-stained cells. The intensity of the MNase sensitivity results was analyzed by densitometry using ImageJ software (v1.8.0).
Data analysis	We took the GRch38 genome (accession codes：GCF_000001405.39 and their associated URL: https://ftp.ncbi.nlm.nih.gov/genomes/all/GCF/000/001/405/GCF_000001405.39_GRCh38.p13/GCF_000001405.39_GRCh38.p13_genomic.fna.gz https://ftp.ncbi.nlm.nih.gov/genomes/all/GCF/000/001/405/GCF_000001405.39_GRCh38.p13/GCF_000001405.39_GRCh38.p13_genomic.gff.gz) as the reference genome for the RNA-seq analysis. PTMap was used to analyze the mass shift 105.0102.EdgeR (v3.6.8) package method was used for screening differentially expressed genes. GSEA analysis of KEGG pathway was implemented using the Diamond (v0.8.33) package. GraphPad Prism (v8.0) were used for data analysis and curve-fitting. Sequencing reads were aligned to the reference genome sequence using Tophat2 (v2.1.1) and bowtie2 (v2.2.2) in default parameter. Genes and isoforms expression level are quantified by a software package: RSEM (RNA-Seq by Expectation Maximization v1.3.0). RSEM computes maximum likelihood abundance estimates using the Expectation-Maximization (EM) algorithm as its statistical model. EdgeR (v3.6.8) package method was used for screening differentially expressed genes.

For manuscripts utilizing custom algorithms or software that are central to the research but not yet described in published literature, software must be made available to editors and reviewers. We strongly encourage code deposition in a community repository (e.g. GitHub). See the Nature Research guidelines for submitting code & software for further information.

Data

Policy information about availability of data

All manuscripts must include a data availability statement. This statement should provide the following information, where applicable:

- Accession codes, unique identifiers, or web links for publicly available datasets
- A list of figures that have associated raw data
- A description of any restrictions on data availability

The datasets generated in the current study are available in the GEO repository under the accession number GSE168473. The mass spectrometric data have been deposited to the ProteomeXchange Consortium with the dataset identifier PXD025490. All data are available in the main text or the Extended Data materials. Source data are provided with this paper.

Field-specific reporting

Please select the one below that is the best fit for your research. If you are not sure, read the appropriate sections before making your selection.

☒ Life sciences ☐ Behavioural & social sciences ☐ Ecological, evolutionary & environmental sciences

For a reference copy of the document with all sections, see nature.com/documents/nr-reporting-summary-flat.pdf

Life sciences study design

All studies must disclose on these points even when the disclosure is negative.

Sample size	Sample size of cell experiments was determined based on previous studies by our group and are similar to those used by other groups (PMIDs 25800736, 28429772,30154464). And no statistical method was used to predetermine sample size in animal studies. The sample sizes used were adequate to obtain clear results based on the magnitude and consistency of measurable differences between experimental groups. For each animal experiments, the number, such n = 10, has been indicated with each figure in the manuscript.
Data exclusions	Data were not excluded from analysis.
Replication	All biological data were confirmed with multiple replicates as noted in the methods and figure legends.
Randomization	Yes, all the animal were randomly to be grouped in all the experiments. To implement random assignment, we assigned a unique number to every member of our study's sample. Then, we used a random number generator to randomly assign each number to a control or INH group.
Blinding	All the bioinformatic and statistical analyses were unbiasedly analyzed or confirmed.The investigators were blinded to group allocation during data collection and/or analysis for bioinformatic and statistical analyses and array experiments. All the array experiments were unbiasedly conducted and analyzed by the sequencing company. All real-time qPCR experiments were performed in a blinded manner. The scoring of the results of immunohistochemistry experiments is done by pathologist, and the researcher is unaware of the sample informations during the scoring process.

Reporting for specific materials, systems and methods

We require information from authors about some types of materials, experimental systems and methods used in many studies. Here, indicate whether each material, system or method listed is relevant to your study. If you are not sure if a list item applies to your research, read the appropriate section before selecting a response.

Materials & experimental systems

n/a	Involved in the study	
☐	☒	Antibodies
☐	☒	Eukaryotic cell lines
☒	☐	Palaeontology and archaeology
☐	☒	Animals and other organisms
☐	☒	Human research participants
☒	☐	Clinical data
☒	☐	Dual use research of concern

Methods

n/a	Involved in the study	
☒	☐	ChIP-seq
☒	☐	Flow cytometry
☒	☐	MRI-based neuroimaging

Antibodies

Antibodies used	Rabbit-pan-Kbz (PTM-762) was purchased from PTM Biolabs: WB,1:1000. Mouse-pan-Kac (sc-32268) were purchased from Santa

	Cruz, Rabbit-pan-Kac (9441S):IF,1:200.Rabbit-total histone H3 (9715S), Rabbit-CBP (7389), Rabbit-P300 (86377), Rabbit-mTOR (2972S), Rabbit-p-mTOR (Ser2448) (2971S), Rabbit-Akt (9272S), Rabbit-p-Akt (Ser473) (9271T) antibodies were purchased from Cell Signaling Technology:WB,1:1000.Rabbit-anti-pan-Kinic antibody was customer designed and prepared by PTM Biolabs Inc. (China): WB,1:1000;IF,1:200.Mouse-anti-Flag (F1804) and Rabbit-anti-HA (H3663) antibodies were purchased from Sigma-Aldrich:WB,1:2000. IF,1:400.Mouse-β-actin (66009-1-lg) and Mouse-GAPDH antibodies (60004-1-lg) were purchased from Proteintech: WB,1:3000.Mouse-anti-PIK3R1 antibody (EM1701-62) was purchased from Huabio (China):WB,1:1000. Rabbit-HDAC3 antibody (T55595) was purchased from Abmart (China): WB,1:1000.12.
Validation	All antibodies were used for applications validated by antibody suppliers per quality assurance provided by each supplier. Most of the antibodies can be validated in the published studies, listed by PMID numbers.<br>Rabbit-pan-Kbz (PTM-762, PMID: 30154464) was purchased from PTM Biolabs: validated from manufacturer's website and citations therein.<br>Mouse-pan-Kac (sc-32268, PMID: 34079475) were purchased from Santa Cruz: validated from manufacturer's website and citations therein.<br>Rabbit-pan-Kac (9441S, PMID: 34158490), Rabbit-total histone H3 (9715S, PMID: 34099795), Rabbit-CBP (7389, PMID: 34149923), Rabbit-P300 (86377, PMID: 34185411), Rabbit-mTOR (2972S, PMID: 34177964), Rabbit-p-mTOR (Ser2448) (2971S, PMID: 34007320), Rabbit-Akt (9272S, PMID: 33258389), Rabbit-p-Akt (Ser473) (9271T, PMID: 33938394) antibodies were purchased from Cell Signaling Technology: validated from manufacturer's website and citations therein.<br>Mouse-anti-Flag (F1804, PMID: 27630089) and Rabbit-anti-HA (H3663, PMID: 31399562) antibodies were purchased from Sigma-Aldrich: validated from manufacturer's website and citations therein.<br>Mouse-β-actin (66009-1-lg, PMID: 29133412) and Mouse-GAPDH antibodies (60004-1-lg, PMID:30293547) were purchased from Proteintech: validated from manufacturer's website and citations therein.<br>Mouse-anti-PIK3R1 antibody (EM1701-62) was purchased from Huabio (China): validated from manufacturer's website and citations therein. Rabbit-HDAC3 antibody (T55595) was purchased from Abmart (China): WB,1:1000. Rabbit-anti-pan-Kinic antibody was customer designed and prepared by PTM Biolabs Inc. (China): validated by dot-blot, WB and IF assays.

Eukaryotic cell lines

Policy information about cell lines

Cell line source(s)	Sf-9 , HepG2, HeLa, HEK293T, HCT-116, HT-29, NCI-H157, NCI-H1299, MEF, HK-2, MCF-7, SUM159, SW1116, HEK293A, MCF-10A (Obtained from ATCC)
Authentication	Sf-9 , HepG2, HeLa, HEK293T, HCT-116, HT-29, NCI-H157, NCI-H1299, MEF, HK-2, MCF-7, SUM159, SW1116, HEK293A, MCF-10A cell lines have not been subjected to additional authentication.
Mycoplasma contamination	The cell lines were tested for mycoplasma contamination and they were mycoplasma-free.
Commonly misidentified lines (See ICLAC register)	Sf-9 , HepG2, HeLa, HEK293T, HCT-116, HT-29, NCI-H157, NCI-H1299, MEF, HK-2, MCF-7, SUM159, SW1116, HEK293A, MCF-10A are not listed as mis-identified cell lines in that database.

Animals and other organisms

Policy information about studies involving animals; ARRIVE guidelines recommended for reporting animal research

Laboratory animals	These mice are on the C57BL/6 background (7–8 weeks old, male, n=10 for each group) and were maintained under a standard 12h dark/light cycle with water and chow diet ad libitum, and temperatures of 65-75°F (~18-23°C) with 40-60% humidity,and were oral gavaged with normal saline or 50 mg/kg/day concentration of INH for ten days, and the mice were sacrificed and liver tissues were harvested for further analysis. All above studies related to animals were approved by the Peking University Health Science Center Institutional Animal Care and Use Committee.
Wild animals	The study did not involve wild animals.
Field-collected samples	The study did not involve field-collected samples
Ethics oversight	All above studies related to animals were approved by the Peking University Health Science Center Institutional Animal Care and Use Committee.

Note that full information on the approval of the study protocol must also be provided in the manuscript.

Human research participants

Policy information about studies involving human research participants

Population characteristics	A total of ten liver cancer patients were recruited among the hospitalized patients. Patients were of both sexes at the age of 25 to 70 years.
Recruitment	Ten paired liver cancer samples were obtained from Peking University People's Hospital from September 2018 to June 2020.
Ethics oversight	All samples were obtained with informed consent at the Peking University People's Hospital. All samples were obtained with informed consent at the Peking University People's Hospital. The studies were approved by the Peking University Health Science Center Ethics Committee under IRB00001052-12088, in accordance with the declaration of Helsinki.

Note that full information on the approval of the study protocol must also be provided in the manuscript.
